# “Modernized” en Bloc Radical Cystectomy Versus Standard Radical Cystectomy: A Nationwide Multi-Institutional Propensity Score Matched Analysis

**DOI:** 10.3390/cancers17030404

**Published:** 2025-01-25

**Authors:** Eirik Kjøbli, Erik Skaaheim Haug, Øyvind Salvesen, Christian Arstad, Anne Kvaale Bergesen, Bjørn Brennhovd, Birgitte Carlsen, Bita Gharib-Alhaug, Gigja Gudbrandsdottir, Patrick Juliebø-Jones, Julie Nøss Haugland, Ann-Karoline Karlsvik, Magnus Larsen, Gunder Magne Lilleaasen, Stig Mûller, May Lisbeth Plathan, Marius Roaldsen, Ingunn Roth, Bernd Lukas Luca Schwenke, Rolf Wahlqvist, Nicolai Wessel, Arne Wibe, Christian Beisland

**Affiliations:** 1 Department of Urology, St. Olav’s University Hospital, 7030 Trondheim, Norway; ann-karoline.karlsvik@stolav.no; 2 Department of Urology, Vestfold Hospital Trust, 3103 Tønsberg, Norway; erik.haug@siv.no (E.S.H.); may.lisbeth.plathan@siv.no (M.L.P.); 3 Department of Clinical Medicine, University of Bergen, 5009 Bergen, Norway; carlsen.birgitte@siv.no (B.C.); charles.patrick.warburton.jones@helse-bergen.no (P.J.-J.); ingunn.roth@helse-bergen.no (I.R.); christian.beisland@helse-bergen.no (C.B.); 4 Department of Clinical and Molecular Medicine, Norwegian University of Science and Technology (NTNU), 7030 Trondheim, Norway; oyvind.salvesen@ntnu.no (Ø.S.); arne.wibe@ntnu.no (A.W.); 5 Department of Urology, Akershus University Hospital, 1478 Nordbyhagen, Norway; christian.arstad@ahus.no (C.A.); gunder.magne.lilleaasen@ahus.no (G.M.L.); stig.muller@ahus.no (S.M.); bernd.lukas.luca.schwenke@ahus.no (B.L.L.S.); 6 Department of Urology, Haukeland University Hospital, 5009 Bergen, Norway; anne.kvale.bergesen@helse-bergen.no (A.K.B.); gigja.gudbrandsdottir@helse-bergen.no (G.G.); julie.noss.haugland@helse-bergen.no (J.N.H.); 7 Department of Urology, Oslo University Hospital, 0586 Oslo, Norway; bjorb@ous-hf.no (B.B.); b26830@ous-hf.no (B.G.-A.); rolwah@ous-hf.no (R.W.); niwessel@online.no (N.W.); 8 Department of Pathology, Vestfold Hospital Trust, 3103 Tønsberg, Norway; 9 Department of Urology, Tromsø University Hospital of North Norway, 9019 Tromsø, Norway; marmagnus.larsen@unn.no (M.L.); marius.roaldsen@unn.no (M.R.); 10 Institute for Clinical Medicine, University of Oslo, 0372 Oslo, Norway; 11 Department of Gastrointestinal Surgery, St. Olav’s University Hospital, 7030 Trondheim, Norway

**Keywords:** bladder cancer, cystectomy, muscle invasive, pelvic lymph node dissection, en bloc, survival, outcome

## Abstract

**Background**: Pelvic lymph node dissection during standard radical cystectomy (stdRC) for muscle invasive bladder cancer is performed as separate templates. In the modernized en bloc radical cystectomy (mEbRC), the bladder is removed together with all its associated lymphatic tissue as one specimen. Our aim was to evaluate the oncological and surgical outcomes of mEbRC with a propensity-matched national cohort of stdRC cases. **Methods**: 935 patients (mEbRC: 214 and stdRC: 721) were eligible for analysis, and 1:2 propensity score matching was performed regressing mEbRC treatment on the variables age, gender, neoadjuvant chemotherapy, Charlson Comorbidity Index, lymph node metastases at final pathology, carcinoma in situ, and pT-stage. The primary outcome was recurrence-free survival (RFS). Secondary endpoints were overall survival (OS) and cancer-specific survival (CSS), survival for female patients. and perioperative measures. **Results**: There were no significant differences between the groups regarding complications, 30-day readmission rates, and 30- and 90-day mortality rates. In the propensity score matched groups, the 5-year RFS was 83% in the mEbRC group vs. 67% in the stdRC group (*p* < 0.001), the CSS was 89% and 78% (*p* ≤ 0.001), and OS 81% vs. 68% (*p* < 0.001) in the same groups, respectively. The results were confirmed by Cox regression analyses with hazard ratios ranging from 0.41 to 0.50 and *p*-values ≤ 0.001, favoring mEbRC. The 5-year OS for female patients was 86% for mEbRC and 60% for stdRC (*p* = 0.022). **Conclusions**: Performing mEbRC over stdRC might yield significantly better oncological outcomes, with equal survival rates for both genders.

## Simple Summary

Despite the advances in medical technology over the last decades, there have only been minor improvements in survival after surgical removal of the bladder for advanced stages of bladder cancer. The investigators suggest that applying the principles of oncological surgery, i.e., extracting the embryonic area of the tumor–affected organ in one specimen and performing an en bloc approach, improves survival. This national Norwegian study included 935 patients treated with either the prior standard technique (n = 721) or this novel en bloc technique (n = 214). The outcome analyses included statistical models reducing possible biases influencing the differences in survival comparing the two groups. The findings indicate that the en bloc technique might yield significantly improved oncologcal outcomes.

## 1. Introduction

Radical cystectomy (RC) with pelvic lymph node dissection (PLND) remains the cornerstone treatment of muscle-invasive bladder cancer (MIBC) [1]. Adding neoadjuvant chemotherapy (NAC) improved survival [2,3], but recent advancements in surgical techniques and technology [4], have yet to demonstrate superior oncological outcomes [5].

Lymph node dissection (LND) during uro-oncological surgery, including RC, is normally performed as a separate template-based step to the removal of the tumor-affected organ. Significant effort has been invested in determining whether PLND should follow a standard or extended template. Two randomized trials [6,7] have failed to show a survival benefit associated with the latter. The debate on PLND during RC has primarily focused on anatomical extent rather than surgical technique. Survival outcome data have been inferior for females, following the aforementioned standard radical cystectomy (stdRC) [8,9]. Superior staging will predict survival or the possibility of dying from the disease according to the treatment, whilst the superior oncological technique results in the best odds for survival. In cases where a complete lymphadenectomy is performed, the number of lymph nodes found in the specimen correlates with the thoroughness of the autopsy technician.

En bloc radical cystectomy (EbRC) was introduced by Skinner in the 1970s and refers to en bloc removal of the bladder together with the associated lymphatic tissue as one specimen [10,11]. It was abandoned around the millennium due to research highlighting the detection of fewer lymph nodes (LNs) compared to when removal was performed as separate packets [12,13]. Inspired by improvements reported in other cancers, where en bloc removal of the embryonic anatomical area of the tumor-affected organ together with intact adherent lymphovascular structures led to better survival rates [14,15], the EbRC method was revisited. Kjøbli et al. developed a modernized version, referred to as mEbRC, which utilizes a similar template and incorporates a non-touch technique [16] as well as sharp dissection along the boundaries using modern surgical equipment. Early results from a single-center study revealed promising improvements in recurrence-free (RFS) and overall survival (OS) [17]. The aim of this study was to compare oncological outcomes from a mEbRC cohort containing all cystectomy patients within a healthcare region over a 7-year period with a comparable national cohort undergoing stdRC.

## 2. Methods

### 2.1. The Bladder Cancer Pathway in Norway

In 2015, the Norwegian Directorate of Health introduced a national healthcare plan for cancer workups called ’Cancer Pathways’ [18]. This included pathways, which mandated that patients at risk of cancer were to be assessed and treated within specific time limits. In Norway, the number of bladder cancer (BC) patients treated outside the public healthcare system is negligible. It is mandatory for patients with BC who are candidates for RC ± NAC to be evaluated by a multidisciplinary team (MDT). This approach ensures that each patient receives a comprehensive evaluation of their clinical status, imaging, histological results, and indication for NAC. It helps formulate an optimal treatment plan, tailored to each patient’s specific needs, adhering to the guidelines.

Given Norway’s challenging geography and a population of approximately 5.5 million with a dispersed settlement pattern, RC is centralized to seven tertiary referral centers. The national guidelines for BC outline the minimum follow-up after RC, which ensures consistent follow-up practice across all centers.

This retrospective study included two cohorts. Cohort A consisted of 224 consecutive patients treated with mEbRC at St. Olavs Hospital, Trondheim, Norway, between January 2017 and June 2023. Implementation of mEbRC was approved by the regional ethics committee for the Central Norway Regional Health Authority (2019/236/REK midt), and all patients signed a written consent form. Cohort A consists of all patients eligible for radical treatment for BC from the Central Norway Regional Health Authority within this timeframe, except for 6 patients receiving trimodal therapy. Cohort B, a national cohort of all 738 consecutive patients undergoing standard RC at six tertiary centers in Norway between 2015 and 2017. These hospitals performed a median of 44.5 (IQR 30–51) cases per year by dedicated cystectomy surgeons.

Inclusion criteria covered patients undergoing RC for BC, encompassing all variant histologies. Twenty-seven patients (2.8%) were excluded: 10 (4.5%) from Cohort A and 17 (2.3%) from Cohort B. Exclusion criteria were: T4b tumors, tumors extending through the peritoneum, metastases at the time of surgery, and patients with suspicious preoperative findings identified as metastases within six months. The differences in exclusions were not statistically significant (*p* = 0.10). The final cohorts consisted of 214 patients in Cohort A and 721 patients in Cohort B. Data collection was completed in February 2024.

StdRC was performed as cystoprostatectomy in men and anterior exenteration in females. The surgical method for Cohort B followed the principles outlined by D’Andrea et al. [19]. The extent of PLND does vary in the material. However, the classification described in the European Association of Urology (EAU) guidelines (limited, standard, extended, and super-extended) has been applied [1]. MEbRC uses the same anatomical landmarks as stdRC in defining the PLND. The primary principle for mEbRC is to mobilize the LN specimen and the bladder as completely in one unit and as intact as possible. The surgical technique for mEbRC has been described previously [17]. In Cohort A, 91.6% of the procedures were performed using a robot-assisted technique, and in Cohort B, 82.6% were treated with open surgery.

### 2.2. Outcomes

The primary outcome of interest was RFS. Secondary endpoints were OS and cancer-specific survival (CSS), female survival, surgical outcomes, and perioperative complications and mortality rates. Treatment of recurrences was regarded as an exploratory endpoint. Recurrence was defined as any recurrence connected to BC treatment with RC, including metastases located in the pelvis, carcinomatosis, recurrence at the ureteroileal anastomosis, LN metastases, or any distant metastases. If possible, recurrences were verified by biopsy or at least CT and/or magnetic resonance imaging. All endpoints were measured from the date of surgery to the date of event. Cause of death was retrieved from the medical journal. If missing, patients with high metastatic burden were considered dead due to BC. Overall mortality is influenced by comorbidity, complications, and treatment of recurrences. The latter were collected and registered as palliative/none, radiation, surgical, chemotherapy, or immunotherapy. In the case of significant differences, all mEbRC patients receiving the treatment were evaluated for a possible effect influencing the outcome.

### 2.3. Statistics

Propensity score matching (PSM) was performed with regressing mEbRC treatment on the following variables: age, gender, NAC, Charlson Comorbidity Index (CCI), LN metastases at final pathology (pN+), carcinoma in situ (CIS), and pT-stage (UICC, Union for International Cancer Control, eighth edition [20]). Every mEbRC patient was matched with the two stdRC patients having the nearest propensity as long as both propensity and deviances were less than the caliper 0.05. Kaplan–Meier analyses and log-rank tests were conducted on the PSM data. Patient characteristics for the matched groups were summarized using the median for continuous variables and proportions for categorical variables. Differences between the groups were examined by using the Mann–Whitney U test for continuous variables and Fisher’s exact test for categorical variables. Multivariable Cox regression analyses were performed on the curative intent-treated group (214 mEbRC vs. 721 stdRC), adjusting for age, gender, neoadjuvant chemotherapy, CCI, LN metastases (pN+), CIS, and pT-stage (UICC). Hazard ratios (HRs) for RFS, CSS, and OS are presented under results, and the complete analyses are available in Supplement A *p*-values < 0.05 were considered statistically significant. Statistical analyses were performed by using R version 3.6.3 and SPSS version 29.0.1.

## 3. Results

### 3.1. Inclusion, Baseline, and Matching

A total of 935 patients (214 mEbRC and 721 stdRC) were eligible for analysis. Baseline characteristics for all patients in both cohorts and PSM groups are presented in Table 1. No significant differences were found for age, gender, CCI, BMI, neoadjuvant chemotherapy, CIS, clinical N-stage, pT-stages, and bladder cancer subtypes for all patients in both cohorts before PSM.

Before PSM, Cohort A had a significantly lower rate of LN metastases (pN+) compared to Cohort B (*p* = 0.03). After 1:2 PSM (195 mEbRC vs. 390 stdRC), the matched parameters showed *p*-values ranging from 0.43 to 1.0. Additionally, non-matched parameters such as BMI, clinical N-stage, and tumor subtype showed *p*-values ≥ 0.2 in the PSM groups. NAC was administered to 49% (76/154) of the mEbRC and 41% (218/530) of the stdRC patients with muscle-invasive cancer in the non-matched groups.

### 3.2. Surgical and Perioperative Outcomes

Table 2 presents perioperative outcomes for the PSM groups. PLND was completed according to the EAU guidelines in 100% of the mEbRC group and 96% of the stdRC group. The incidence of positive surgical margins at final pathology was higher in the stdRC group compared to the mEbRC group, at 4.9% vs. 0.5% (*p* < 0.001). Estimated blood loss was lower in the mEbRC group than in the stdRC group, with 300 mL vs. 400 mL, respectively (*p* < 0.001). Urinary reconstruction using an ileal conduit was chosen for 85% of the mEbRC patients and 89% of the stdRC patients in the PSM groups. There were no significant differences between the mEbRC and stdRC groups regarding complications according to the Clavien-Dindo classification, 30-day readmission rates, and 30- and 90-day mortality rates (Table 2).

### 3.3. Recurrence-Free, Cancer-Specific, and Overall Survival

**Figure 1:**
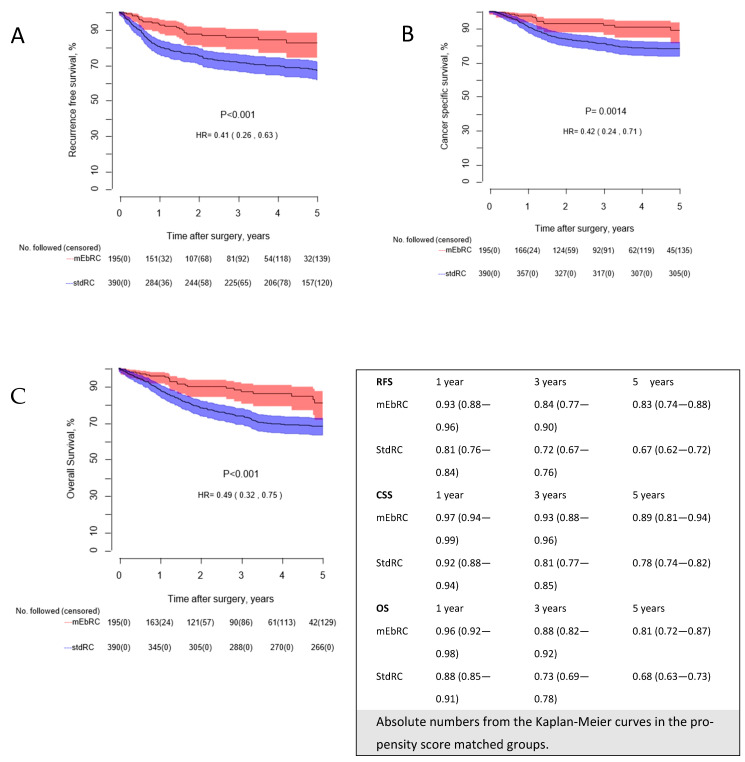
(**A**–**C**): Kaplan–Meier curves comparing (**A**): recurrence-free survival, (**B**): cancer-specific survival, and (**C**): overall survival, for patients in the propensity score matched groups. The number of patients followed without an event in each group are reported annually with censored in parenthesis. mEbRC = modernized en bloc Radical Cystectomy, StdRC = standard Radical Cystectomy.

Figure 1A–C presents the Kaplan–Meier estimates for RFS, CSS, and OS in the PSM groups. The mEbRC group showed statistically significantly better outcomes across all three survival analyses compared to the stdRC group (all *p*-values ≤ 0.001, with HRs ranging from 0.41 to 0.49).

**Figure 2:**
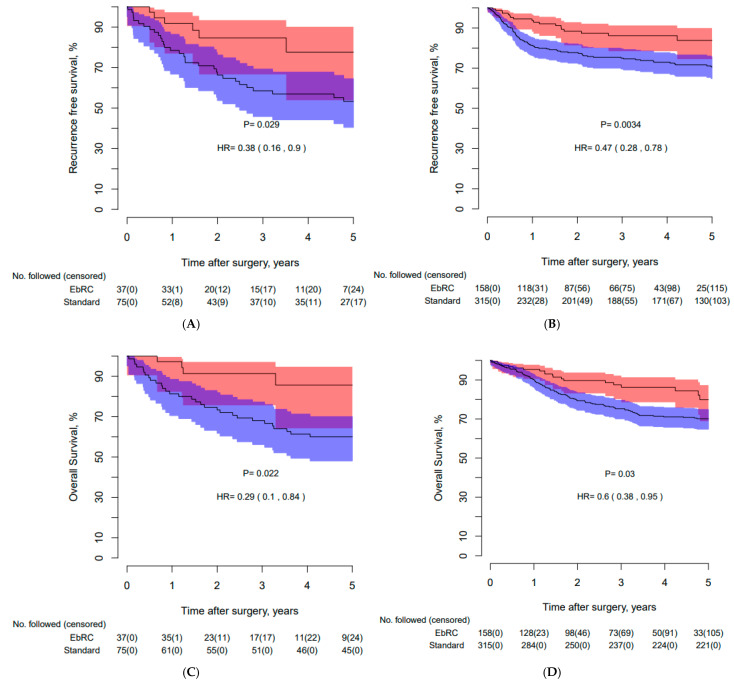

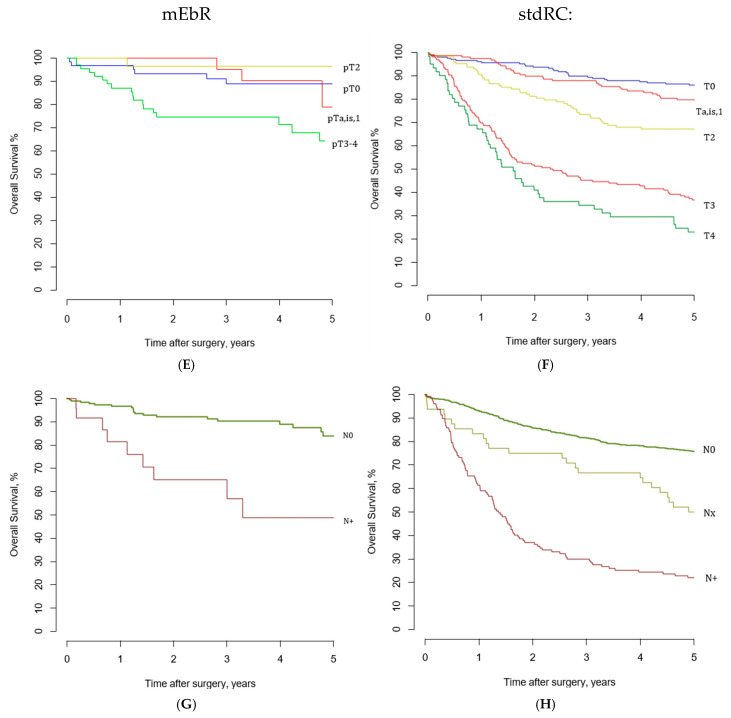

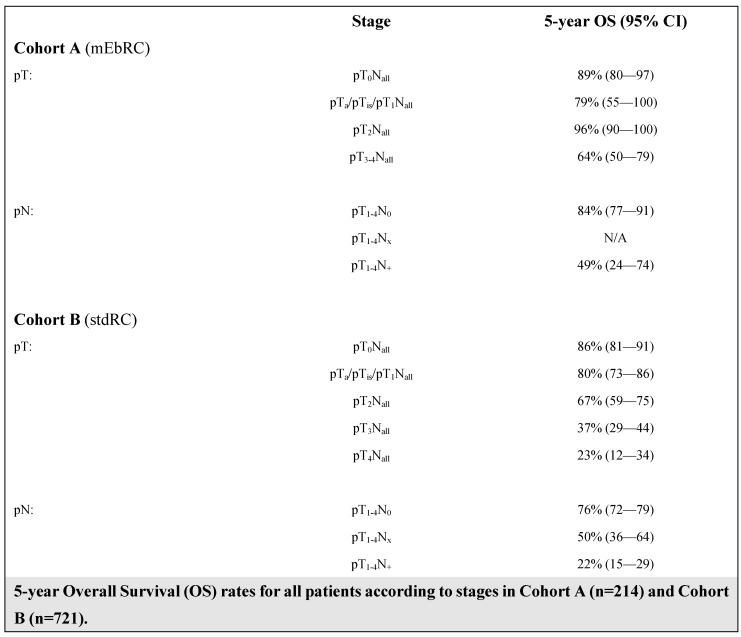
Kaplan–Meier curves presenting (**A**): recurrence-free survival for females in the PMS groups, (**B**): recurrence-free survival for males in the PMS groups, (**C**): overall survival for females in the PMS groups, (**D**): overall survival for males in the PMS groups (**E**): overall survival for all mEbRC patients according to pT-stages, (**F**): overall survival for all stdRC patients according to pT-stages, (**G**): overall survival for all mEbRC patients according to pN-stages, and (**H**): overall survival for all stdRC patients according pN-stages. PSM: propensity score matched, pT-stage: pathological stage at final pathology, pathological N-stage is defined as N+ or N0; mEbRC does not allow the differentiation between N1 to N3, Nx: Lymph node status not reported in final pathology or pelvic lymph node dissection not performed, mEbRC: modernized en bloc radical cystectomy, stdRC: standard radical cystectomy.

Kaplan–Meier curves in Figure 2A–D present similar survival outcomes for female patients compared to male patients, and a larger absolute OS benefit compared to stdRC (86% EbRC vs. 60% stdRC, *p* = 0.02). The 5-year OS data per pT-stage and pN-status for both Cohorts are presented in Figure 2E–H.

A Cox proportional hazard regression model, applied to all curative intent-treated patients and adjusted for the same parameters used for PSM, yielded the following HRs for mEbRC vs. stdRC: 0.41 (CI: 0.27–0.61, *p* ≤ 0.001) for RFS, 0.44 (CI: 0.27–0.71, *p* ≤ 0.001) for CSS, and 0.50 (CI: 0.34–0.73, *p* ≤ 0.001) for OS. Complete analyses are provided in Supplement A. Kaplan–Meier estimates for 5-year RFS, CSS, and OS for all patients in both cohorts are presented in Supplement B.

### 3.4. Treatment of Recurrent Disease

Table 3 shows treatments given for recurrences in the PSM groups. More patients received palliative care or surgery in the stdRC group, while more patients in the mEbRC group received immunotherapy. Seven of the eight mEbRC patients treated with immunotherapy received 1 to 7 rounds of treatment with no effect, and one patient received 16 rounds. The patient receiving 16 rounds died 21 months after being diagnosed. None of the patients were long-term survivors.

## 4. Discussion

The main finding is that mEbRC significantly improved RFS, CSS, and OS compared to stdRC. The key difference between these techniques lies in the different approach to PLND. This modification to surgical technique requires no additional equipment and incurs no extra financial costs.

Prior studies have shown less favorable outcomes for females [8,9]. To our knowledge, this is the first cystectomy technique where the female patients seem to benefit the most. The findings are supported by the results in the annual bladder cancer report from the Cancer Registry of Norway [21] (patients treated between 2019 and 2023; Supplement C). A possible explanation for the effect observed among females is the difference in the proximity to the resection margin and the partition of the lymphovascular structures. However, additional research is needed to explore this further.

PLND in RC is debated, including whether it should be primarily a staging tool, a therapeutic procedure, or both. The EAU guidelines [1] support a template-based standard PLND, which effectively identifies almost all node-positive patients due to the rarity of skip metastases. Most surgeons prefer to retrieve the PLND specimens using separate templates, as this has been reported to result in a higher LN yield and superior staging [22]. There is less agreement regarding the therapeutic value of PLND. Retrospective studies suggest better outcomes with PLND compared to without [23], which are improved when an extended approach is adopted in favor of limited PLND. However, randomized trials have failed to show improved survival with extended over standard LND [6,7].

The rationale for a therapeutic effect of PLND includes the removal of micrometastases, reduction in local recurrence, and therapeutic benefits for node-positive disease [24]. In other cancers (e.g., esophagus and rectal), new techniques have been developed, revealing that implementing en bloc removal of the embryonic anatomical area of the tumor-affected organ together with the adherent lymphovascular structures leads to improved oncological outcomes [14,15]. The early EbRC methods described by Skinner and Stein were perhaps hampered by the surgical technique of the time, resulting in disease recurrences. We believe that modern surgical technology, thorough preoperative planning, and the non-touch technique, in combination with complete en bloc removal of the bladder and LNs, increase the therapeutic effect. No evidence in modern oncological surgery was found supporting superior or equal outcomes for template-based over en bloc lymphadenectomy for aggressive cancer [14,15,25,26,27]. The best possible staging provides a more accurate prediction of outcome, but actual survival is related to the surgical technique. This might be the reason why randomized trials [6,7] have failed to prove survival benefits for extended lymphadenectomy.

In the absence of a randomized trial, we employed PSM to balance the mEbRC and the stdRC groups. PSM reduces the risk of selection bias and unknown confounders; however, factors not adjusted for in the PSM process may still influence the outcome. After PSM, the two groups were statistically similar not only for the matched parameters but also for BMI, clinical N-stage, and tumor subtype, indicating a general similarity in the baseline characteristics. This is not unexpected, as the cohorts included all patients offered RC under the same regulations and guidelines. To confirm survival outcomes, we applied Cox multivariable regression analyses, and the favorable outcomes for mEbRC persisted with very similar HRs.

When comparing perioperative outcomes, there were no significant differences between the two groups. The presented data also align with previously publications for perioperative complications, readmissions, and 30- and 90-day mortality [23,28,29]. A 4.9% positive surgical margin rate in the stdRC group is similar to previous reports [4,28,30]. The lower positive margin rate for mEbRC is likely a result of the mEbRC technique rather than the level of surgical expertise.

Another factor not adjusted for is the distinction between open and minimally invasive techniques. The difference in estimated perioperative blood loss is consistent with earlier data and is likely due to the robot-assisted technique mostly used in the mEbRC group. Randomized trials have failed to demonstrate any superior oncological outcomes after robot-assisted radical cystectomy [4], making a clinically significant impact less probable. The present amount of minimally invasive surgery is higher, but still about 50% of cystectomies in Norway are performed open. All cystectomies in Norway are treated at tertiary centers by dedicated cystectomy surgeons performing > 10 cases/years. It is therefore unlikely that the outcomes are hampered by inter-center variability in surgical expertise.

The complexity of the caseload, particularly the distribution of comorbidity and tumor stage, influences surgical and oncological outcomes. It might pose a problem when discussing single-center series. In such a nationwide study, such bias is lessened. Public healthcare systems with national cancer pathways and guideline-driven MDTs support consistent treatment recommendations. This was the case in the present study, where despite covering different timeframes, the cohorts were similar even before matching. Management algorithms remained unchanged during both time periods.

There are studies showing survival data for patients treated with surgery alone [31]. However, modern BC treatment includes NAC. Both of our cohorts demonstrated a high use of NAC. Due to its impact on survival, NAC was included in the PSM [2,3,32].

The difference in RFS, CSS, and OS in favor of mEbRC was significant. This raises the question of whether the survival results in the stdRC cohort are satisfactory. In the stdRC cohort, this study reported a 5-year CSS and OS of 76% and 65%, respectively. This aligns with a recent publication from the Finnish National Cystectomy Database reporting a 5-year CSS and OS of 74% and 66%, respectively [33]. Other contemporary studies also report survival rates as presented for stdRC [13,23,30]. When comparing the present results to those of the Finnish study [33] and examining the per-stage 5-year OS rates for stdRC, similar rates are seen for pT0, pTa/pTis/pT1, and pT2 (Figure 2). However, for pT3, pT4, and pN+, the present results for stdRC are poorer per stage. One reason for this might be the higher use of NAC in our series (>30% vs. <20%). Mitra et al. proposed this phenomenon as a result of NAC non-responders [13]. Of further interest is that the improved OS in pT2, pT3, pT4, and pN+ drives the overall improved survival in the mEbRC group (Figure 2). The group with advanced disease and NAC non-responders might be a subset where the therapeutic effect of PLND is of significant importance and should be explored further.

Recurrence treatments are undergoing changes, which may impact survival outcomes. Immunotherapy was approved in Norway as a second-line treatment in 2018, as a first-line treatment for chemotherapy-ineligible patients in 2022, and as adjuvant therapy in 2023 [34]. Immunotherapy was more frequently administered to patients with recurrence after mEbRC than stdRC (mEbRC 33% vs. stdRC 9%). Of the eight patients receiving immunotherapy for recurrence after mEbRC, seven showed no clinical effect, and all eight died within two years of diagnosis. In our opinion, it is unlikely that this difference affected the overall study outcome.

### Strengths and Limitations

One of the main strengths of this study is its comprehensive data collection, which utilized a large and consecutive national cohort to minimize selection bias and enhance the robustness of the findings. Use of PSM helps balance the cohorts, reduce risk of confounders, and ensure comparability between groups. However, this study is retrospective, and there are accordingly recognized shortcomings. This includes the limitation that it was not possible to adjust for all known risk factors, such as tumor location [35], in the statistical models used. For instance, a high ratio of factors to events in the Cox analyses can lead to the issue of overfitting. MEbRC was only performed at one center. Despite including consecutive data from all centers performing RC in Norway, the differing case volumes, as well as surgical practice patterns, do introduce variability. However, national efforts to implement standardized protocols across Norway have ensured consistent treatment and follow-up, which enhances the reliability of the results. Prospective studies are needed to ratify these findings including a national randomized multicenter trial, which is planned in Norway.

## 5. Conclusions

Implementation of mEbRC might significantly improve RFS, CSS, and OS compared to stdRC, with equal or superior outcomes for female patients.

## Figures and Tables

**Table 1:** Baseline characteristics before and after matching.

	Before Matching					After 1:2 Matching				
	All mEbRC (*n =* 214)		All stdRC (*n* = 721)		*p*-Value	mEbRC Matched (*n* = 195)		stdRC Matched (*n =* 390)		*p*-Value
**Age**	70.9	(64–77)	70.4	(64–75)	0.44	71.5	(65–75)	70.0	(63–74)	0.57
**Gender**					0.93					1.0
**Male**	169	(79%)	565	(78%)		158	(81%)	315	(81%)	
**Female**	45	(21%)	156	(22%)		37	(19%)	75	(19%)	
BMI *	25.9	(24–30)	25.5	(23–28)	0.09	26.2	(24–30)	25.9	(23–29)	0.20
Clinical N-stage *										
**cN+**	16	(8%)	76	(11%)	0.24	13	(7%)	38	(10%)	0.28
**Neoadjuvant chemotherapy**					0.32					1.0
**Yes**	76	(35.5%)	230	(32%)		64	(33%)	129	(33%)	
**No**	138	(64.5%)	491	(68%)		131	(67%)	261	(67%)	
**Charlson Comorbidity Index**										
	0	(0–2)	1	(0–2)	0.44	0	(0–1)	0	(0–1)	0.78
**Pathological N-stage**					<0.001					
**pN+**	24	(11.2%)	127	(17.6%)	0.03	22	(11.3%)	49	(12.6%)	0.69
**pN0**	190	(89%)	546	(76%)		173	(89%)	341	(87%)	
**pNx**			48	(6.7%)						
**Carcinoma in situ**					0.10					0.54
**Yes**	108	(51%)	316	(44%)		92	(47%)	195	(50%)	
**No**	106	(49%)	405	(56%)		103	(53%)	195	(50%)	
**Pathological stage**					0.75					0.43
**pT0**	64	(30%)	208	(29%)	0.80	59	(30%)	118	(30%)	1.0
**pTis**	12	(5.6%)	61	(8.5%)	0.19	12	(6%)	39	(10%)	0.16
**pTa**	11	(5.1%)	26	(3.6%)	0.32	11	(5.6%)	12	(3.1%)	0.17
**pT1**	25	(12%)	71	(10%)	0.44	24	(12%)	42	(11%)	0.58
**pT2**	37	(17%)	128	(18%)	0.92	33	(17%)	59	(15%)	0.63
**pT3**	48	(22%)	166	(23%)	0.93	40	(21%)	93	(24%)	0.40
**pT4a**	17	(7.9%)	61	(8.5%)	0.89	16	(8%)	27	(7%)	0.62

Data are median (IQR) or n (%). Propensity score matching was performed regressing mEbRC treatment on the variables age, gender, neoadjuvant chemotherapy, Charlson Comorbidity Index, lymph node metastases at final pathology (pN+), carcinoma in situ, and pT-stage. Every mEbRC patient was matched with the two stdRC patients having the nearest propensity, provided both propensity deviances were less than the caliper 0.05. mEbRC: modernized en bloc radical cystectomy, stdRC: standard radical cystectomy, pT: pathological stage (final pathology report, Union for International Cancer Control, eighth edition), pathological N-stage is defined as N+ or N0; mEbRC does not allow the differentiation between N1 to N3, Nx: Lymph node status not reported in final pathology or pelvic lymph node dissection not performed. * = Not included in the propensity score matched analyses.

**Table 2:** Perioperative outcomes in the propensity score matched groups.

	After 1:2 Matching				
	mEbRC Matched (n = 195)		stdRC Matched (n = 390)		*p*-Value
**High grade cancer**					1.0
**Yes**	192	(99%)	383	(98%)	
**No**	3	(2%)	5	(1%)	
**N/A**			2	(1%)	
**PLND**					<0.001
**Superextended**			14	(3.6%)	0.007
**Extended**	115	(59%)	90	(23%)	<0.001
**Standard**	80	(41%)	272	(70%)	<0.001
**Limited**			6	(1.5%)	
**None/unknown**			8	(2.1%)	
**Lymph nodes in final pathology**	17	(12–23)	15	(9–20)	<0.001
**Extended**	21	(13–26)	17	(12–21)	0.002
**Standard**	14	(11–19)	14	(9–20)	0.91
**Histologic subtype**					0.66
**Urothelial carcinoma**	186	(95%)	375	(96%)	
**Squamous cell carcinoma**	4	(2.1%)	7	(1.8%)	
**Adenocarcinoma**	2	(1.0%)	4	(1.0%)	
**Small cell carcinoma**			2	(0.5%)	
**Sarcoma**	3	(1.5%)	2	(0.5%)	
**Positive surgical margin**					
**Yes**	1	(0.5%)	19	(4.9%)	<0.001
**No**	194	(99%)	371	(95%)	
**Complications**					
**CL-D 2**	72	(37%)	146	(37%)	0.93
**CL-D 3**	22	(11%)	48	(12%)	0.79
**CL-D 4**	1	(0.5%)	8	(2.1%)	0.28
**Perioperative blood loss (mL)**	300	(150–450)	400	(250–600)	<0.001
**30-day readmission rate**	23	(11.8%)	34	(8.7)	0.24
**Mortality rates**					
**30-day**	1	(0.5%)	4	(1%)	0.67
**90-day**	5	(2.6%)	12	(3.1%)	0.80

Data are median (IQR) or n (%). mEBRC: modernized en bloc radical cystectomy, stdRC: standard radical cystectomy, PLND: pelvic lymph node dissection, Cl-D: Clavien-Dindo classification of complications, N/A: not available.

**Table 3:** Treatment recurrence.

	After 1:2 Matching				
	mEbRC Matched (n = 195)		stdRC Matched (n = 390)		*p*-Value
**Treatment recurrence**	n = 24		n = 116		
**None**	4	(17%)	18	(16%)	1.0
**Palliative**	5	(21%)	55	(47%)	0.02
**Surgery**	0		18	(16%)	0.04
**Radiation**	4	(17%)	39	(34%)	0.14
**Chemotherapy**	12	(50%)	54	(47%)	0.82
**Immunotherapy**	8	(33%)	11	(9%)	0.005

Data are n (%). mEBRC: modernized en bloc radical cystectomy, stdRC: standard radical cystectomy.

## Data Availability

The raw data supporting the conclusions of this article can be made available by the authors on request.

## Supplementary Materials

The following supporting information can be downloaded at: https://www.mdpi.com/article/10.3390/cancers17030404/s1.
