# Supplementary material for: “Modernized” en Bloc Radical Cystectomy Versus Standard Radical Cystectomy: A Nationwide Multi-Institutional Propensity Score Matched Analysis
Source: Cancers (Basel). 2025 Jan 25;17(3):404. doi: 10.3390/cancers17030404 (PMC11816131; doi:10.3390/cancers17030404)
Supplement: Supplementary file 1 [file cancers-17-00404-s001.zip › cancers-3400711-supplementary.pdf]

Supplement A

	HR:	95% CI	p=
**Recurrence-free survival:**			
**Unadjusted mEbRC vs. stdRC**	0.42	0.28—0.63	≤0.001
**Adjusted mEbRC vs. stdRC**	0.41	0.27—0.61	≤0.001
**Age**	1.01	0.99—1.02	0.22
**Gender**	1.27	0.95—1.69	0.10
**Neoadjuvant chemotherapy**	1.15	0.86—1.55	0.35
**CCI**	1.11	1.00—1.25	0.06
**pN+**	5.31	4.03—6.98	≤0.001
**pNx**	1.32	0.72—2.41	0.37
**CIS**	0.81	0.63—1.05	0.11
**pT0-1**	-	—	-
**pT2**	1.02	0.65—1.59	0.93
**pT3-4**	2.78	1.88—4.11	≤0.001
**Cancer specific survival:**			
	HR:	95% CI	p=
**Unadjusted mEbRC vs. stdRC**	0.42	0.26—0.67	≤0.001
**Adjusted mEbRC vs. stdRC**	0.44	0.27—0.71	≤0.001
**Age**	1.02	1.00—1.03	0.07
**Gender**	1.24	0.90—1.70	0.19
**Neoadjuvant chemotherapy**	1.52	1.09—2.12	0.01
**CCI**	1.02	0.91—1.16	0.72
**pN+**	5.85	4.31—7.93	≤0.001
**pNx**	1.20	0.60—2.41	0.60
**CIS**	0.79	0.60—1.06	0.11
**pT0-1**	-	—	-
**pT2**	0.78	0.47—1.32	0.36
**pT3-4**	2.33	1.50—3.63	≤0.001
**Overall survival:**			
	HR:	95% CI	p=
**Unadjusted mEbRC vs. stdRC**	0.49	0.34—0.71	≤0.001
**Adjusted mEbRC vs. stdRC**	0.50	0.34—0.73	≤0.001
**Age**	1.04	1.02—1.05	≤0.001
**Gender**	1.18	0.91—1.52	0.21
**Neoadjuvant chemotherapy**	1.19	0.91—1.57	0.20
**CCI**	1.20	1.09—1.32	≤0.001

**pN+**	4.01	3.12—5.15	≤0.001
**pNx**	1.68	1.09—2.56	0.02
**CIS**	0.84	0.67—1.05	0.13
**pT0-1**	-	-	-
**pT2**	0.80	0.56—1.14	0.22
**pT3-4**	1.96	1.43—2.68	≤0.001

Multivariable Cox regression analysis performed on the curative intent treated group (935 patients; 214 mEbRC and 721 stdRC, of which 4 stdRC patients were excluded due to missing variables) adjusting for age, gender, neoadjuvant chemotherapy, Charlson Comorbidity Index, pN+: lymph node metastases in final pathology report, Nx: Lymph node status not reported in final pathology or pelvic lymph node dissection not performed, CIS: carcinoma in situ, pT: pathological stage (final pathology report, Union for International Cancer Control, eighth edition), mEbRC: modernized en bloc radical cystectomy, stdRC: standard radical cystectomy, HR: Hazard ratio, CI: Confidence interval.

Supplement B: Multivariable Cox regression analyses

Supplement B

A

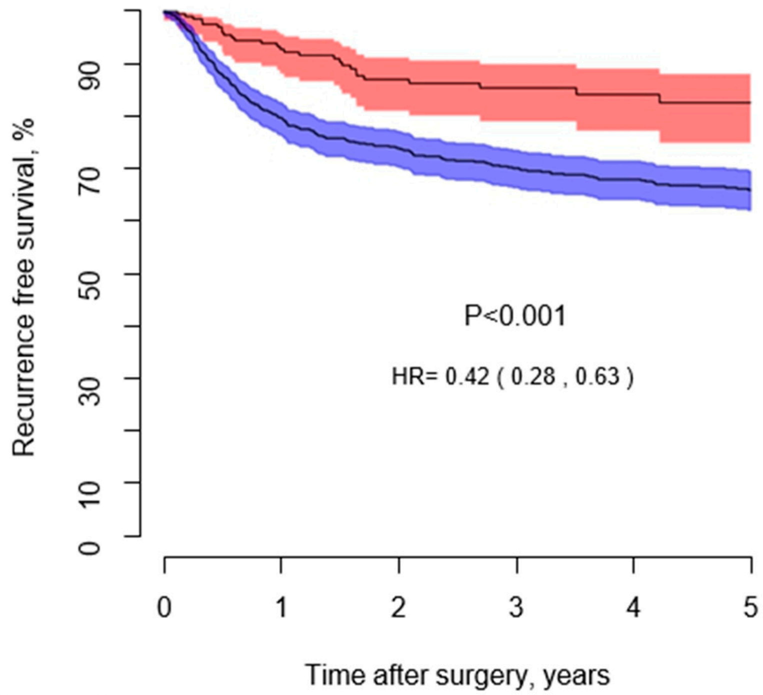

No. followed (censored)						
---mEbRC	214(0)	166(34)	118(73)	91(98)	60(128)	37(150)
---stdRC	721(0)	513(72)	443(105)	402(124)	366(148)	290(214)

B

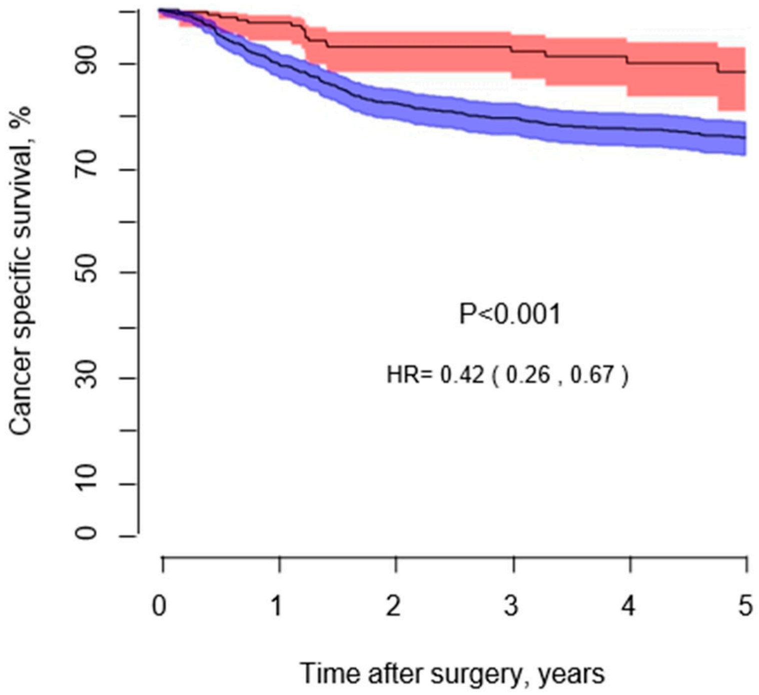

No. followed (censored)						
---mEbRC	214(0)	183(26)	139(62)	105(96)	70(128)	50(147)
---stdRC	721(0)	649(0)	593(0)	573(0)	558(0)	546(0)

C

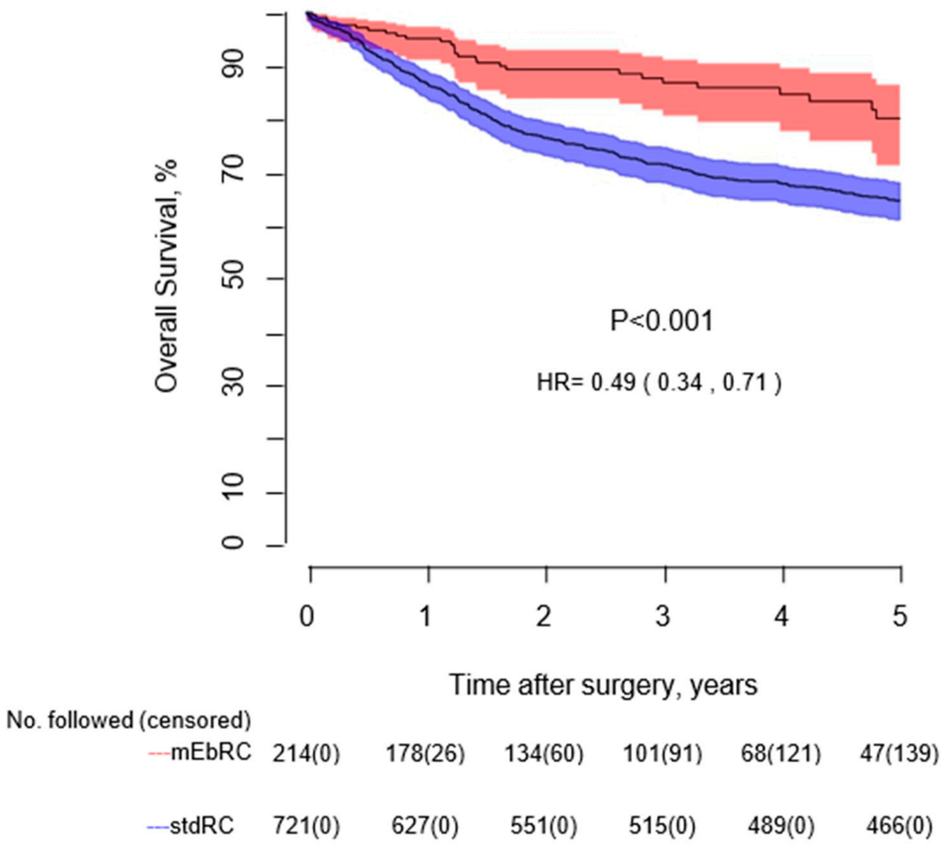

*K-M curves for all patients comparing **A:** recurrence-free survival, **B:** cancer specific survival, and **C:** overall survival. The number of patients followed without an event in each group are reported annually with censored in parenthesis. mEbRC= modernized en bloc radical cystectomy, stdRC= standard radical cystectomy*

**RFS**	1 year	3 years	5 years
mEbRC	0.93 (0.88—0.96)	0.85 (0.79—0.90)	0.82 (0.75—0.88)
stdRC	0.80 (0.77—0.83)	0.70 (0.66—0.75)	0.66 (0.62—0.70)
**CSS**	1 year	3 years	5 years
mEbRC	0.98 (0.94—0.99)	0.93 (0.88—0.96)	0.88 (0.81—0.93)
stdRC	0.90 (0.88—0.92)	0.79 (0.76—0.82)	0.76 (0.72—0.79)
**OS**	1 year	3 years	5 years
mEbRC	0.95 (0.91—0.97)	0.88 (0.82—0.92)	0.80 (0.71—0.86)
stdRC	0.87 (0.84—0.89)	0.71 (0.68—0.75)	0.65 (0.61—0.68)

Absolute numbers for survival in Kaplan-Meier curves for all patients at 1, 3, and 5 years

Supplement C

2.7 Overlevelse

2.7.1 5-års relativ overlevelse for kvinner med muskelinfiltrerende blærekreft (MIBC)

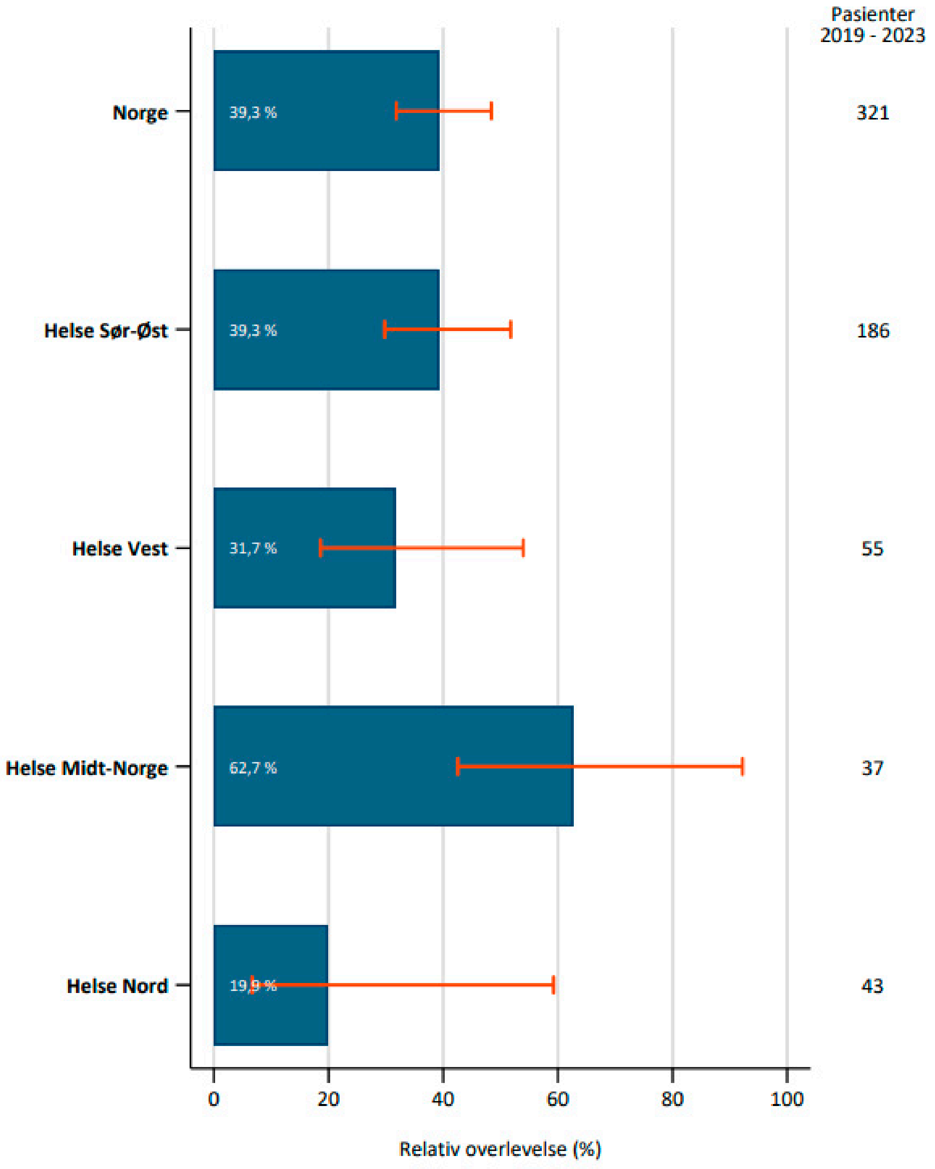

Figur 2.16: 5-års relativ overlevelse for kvinner med muskelinfiltrerende blærekreft (MIBC) fordelt på opptaksområde (bosted) i perioden 2019–2023.

This figure is form the annual 2023 national bladder cancer report from The Cancer Registry of Norway. It presents 5-year relative survival for females with muscle invasive bladder cancer diagnosed from 2018 to 2023 divided in to Norwegian healthcare regions. All female patients in "Helse Midt-Norge" (Middle Norway) were treated with en bloc radical cystectomy. There is a trend towards better outcome, but due to the small sample size and overlapping confidence intervals, more patients and longer observation time is needed to confirm the differences.
